# Online Involvement for Georgia Student Teachers During Covid-19

**DOI:** 10.3389/fpsyg.2021.648028

**Published:** 2021-06-03

**Authors:** Michelle A. Thomas, Martin Norgaard, Laura A. Stambaugh, Rebecca L. Atkins, Anita B. Kumar, Alison L. P. Farley

**Affiliations:** ^1^School of Music, Georgia State University, Atlanta, GA, United States; ^2^Department of Music, Georgia Southern University, Statesboro, GA, United States; ^3^Hugh Hodgson School of Music, University of Georgia, Athens, GA, United States

**Keywords:** preservice teacher, digital learning, Covid-19, distance learning, student teachers, music education, teacher education

## Abstract

As concerns about Covid-19 rapidly escalated in March 2020 in the United States, all levels of education were impacted. A unique population (student teachers) faced challenges from two perspectives: as students and as teachers forced to teach and learn from a distance. Student Teachers, or preservice teachers (PST), are university students finishing a degree and/or teacher certification program by serving as an intern in a school setting. As schools were closed, these PSTs may not have been given access to the online learning platforms of their cooperating teachers (CT) and were no longer included in classroom instruction. The purpose of this study was to examine how the sudden shift away from traditional face-to-face instruction, co-teaching, and mentorship affected the involvement of music PSTs and their CT mentors in one region of the United States. Specifically, the research questions were: (1) How and in what ways were PSTs involved in planning, instruction, and/or assessment synchronously and asynchronously after school closures? (2) In what subdomains (performance, music theory/ear-training, etc.) were PSTs engaged in instruction and learning activities? (3) What challenges and solutions did PSTs report related to Covid-19 closures? A survey was sent, *via* email, to PSTs attending teacher preparation programs at universities in the state of Georgia at the end of the spring semester. Thirty-seven participants responded to the survey questions representing about 32% of all PSTs in Georgia in Spring 2020. Twenty-one were not given access to the online teaching platform of their placement school. A thematic analysis of the open-ended questions identified common themes including whether experiences were perceived as negative or positive. Of the PSTs given access, the majority of their responsibilities and experiences were creating assignments, additional help videos, participating in Zoom meetings, and assessing student assignment submissions. Of these experiences, interestingly, most were classified as positive by the PSTs. However, the importance of face-to-face interactions for both PST and the P-12 students was mentioned throughout survey responses. Approximately 10 PSTs mentioned their CT relationship/interaction and four of the respondents noted that their CT never reached out for help; however, six noted collaborative meetings or teaching with their CT. Importantly, some PSTs reported a lack of knowledge related to the planning and implementation of music instruction in the online modality. Therefore, teacher preparation programs should consider incorporating technology including online solutions into the music curriculum so that future music educators may more flexibly incorporate both in-person and distance learning.

## Introduction

Spring of 2020 will go down in history for how the world pressed pause as a deadly disease traveled the globe. In the United States, where the Covid-19 death toll continued to rise, teachers and parents tried to keep life moving and find some sense of normalcy for the sake of the students and children. Though K-12 schools shut down and universities sent students home, learning had to continue. Teachers had to quickly develop online learning modules to keep students engaged for the rest of the academic year. For preservice music education majors, this meant navigating both the pandemic’s effects on them as students, as well as their field experiences working with master teachers. As faculty advising these students and professionals, we witnessed a variety of individual situations and sought to better understand the broader picture in our state.

As is common in many places, in the United States, the undergraduate music education major leading to state-awarded teacher certification includes required field experiences. These experiences range from observations of classrooms to co-teaching with cooperating teachers (CT), to these preservice teachers (PST) eventually planning, teaching, and assessing students on their own under the guidance and supervision of their CT and a University Supervisor. Students who are in their final semester before graduation and receiving certification are usually engaged in a full-time internship in a local school with a music educator who teaches the subspecialty these PSTs most want to teach upon graduation (for example, high school band, choir, or orchestra; middle school band, choir, or string orchestra, elementary general music, and secondary music technology). However, preservice teacher field experiences also take place before this final semester, sometimes as a part-time internship or other practicum experience. PSTs are, therefore, in the positions of both university student and preservice teacher. As the pandemic shook both K-12 schools and universities, PSTs had to quickly navigate an avalanche of new remote learning technology requirements from both perspectives and develop competencies amid negotiating access for themselves and their students. Overall, we sought to determine: What did this abrupt shift look like for those music PST engaged infield experience at the point of pandemic closures? What was life like for the PST and their CT now balancing distanced digital learning, the pandemic, school, and personal life all while sheltering in place? Furthermore, how did this unique experience demonstrate ways in which the standard field experience model needs to adapt to contemporary needs, including unforeseen challenging circumstances on such a massive social scale?

## Review of Literature

### Preservice Teacher Field Experience

In the United States, PSTs may participate in a variety of field experiences, including observations of teaching in schools, viewing of videos, short-term or one-off practice lessons with K-12 students (“practicum”), and longer-term internships as a culminating experience before receiving certification (“student teaching”). In the state of Georgia, the requirement includes:

Culminating residency (formerly referred to as *student teaching*) or internship experiences with candidates placed in classrooms for at least one (1) full semester where they experience intensive and extensive practices in which they are fully immersed in the learning community and provided opportunities to develop and demonstrate competence in the professional roles for which they are preparing (Georgia Professional Standards Commission, 2020).

Before this culminating residency, many PSTs in Georgia complete shorter-term practice lessons (“practicum”) and several Educator Preparation Programs included a part-time residency in the semester preceding the culminating residency. For the purpose of this article, we will refer to both the part-time and full-time residencies as “student teaching,” as they meet the criteria of intensity, extensity, and immersion outlined above.

Student-teacher field experience often encompasses more observation than actual interactive teaching (Kelly, 2015). Researchers have noted that student teachers need more in-classroom experiences and content knowledge to have the ability to reflect on a deeper level to better increase their teaching quality (Stegman, 2007). Therefore, more Georgia Educator Preparation Programs have implemented a part-time student teaching experience to immediately precede the culminating student teaching semester. Researchers have shown that field experience early and continuously through PST’s professional development is critical to the formation of self-efficacy related to their music teaching skills (Barry and Caravan, 2020). Before participation in their student teaching field experience, PSTs lack the knowledge and experience to teach independently to the best of their ability.

This lack of experience and content knowledge has led teacher education programs to seek alternative student teaching models to better gradually transition PSTs to independent self-regulated teaching (Baeten et al., 2018). The co-teaching model of field experience for PST is becoming a focus of research with hopes of better supporting new teachers to keep them in the field. Co-teaching or team teaching is an alternative to the standard method. Team teaching is defined as two or more teachers collaboratively planning, delivering, and evaluating a class session (Baeten et al., 2018). Co-teaching has the potential to provide a true partnership between higher education and K-12 schools; this approach encourages reciprocal learning between PST and CT (Zach, 2020). The co-teaching approach is not just beneficial to the student teachers, but also to the cooperating teachers who are willing to mentor and learn from the experience of collaboration. Researchers have noted that cooperating music teachers see their co-teaching experience as beneficial to their teaching and their students’ learning (Kim, 2019).

Preservice teachers see their CTs as the role models and the classrooms they contribute to become environments where they see examples of how music teaching and learning should be done (Abrahams, 2011). The relationship formed between PSTs and CTs is important to the development of the PSTs’ success and transition because the PSTs are primarily under the direction and supervision of their CTs who become solely responsible for their in-class and out-of-class experiences (Kelly, 2015). Researchers have noted that the various degrees of supervision may allow the PSTs the opportunity to experiment with pedagogical techniques without fear of consequence along with gaining critical ongoing feedback from their CTs and university supervisors through formal and informal observations (Bartolome, 2017). The CT is the first mentor a new teacher has in their new teacher journey, and a CT’s efforts as a mentor during a PST’s field experience can help better guide their development into independent teaching (Lejonberg et al., 2018).

The student teaching field experience revolves around the PSTs’ involvement in the planning, teaching, and assessment of the K-12 students in their CTs’ classrooms. Researchers focused on PST field experience have noted PSTs’ inability to cite specific teaching strategies they planned to use in their future teaching (Barry and Caravan, 2020). PSTs have frequently been noted as complaining about only getting to observe and not getting enough opportunities for hands-on teaching, causing them to disengage with the classroom activities and often become hypercritical of their CT. This judgment of CTs’ teaching, planning, and assessment techniques triggers a conflict within PSTs. The only experience they have are their own K-12 music classroom involvements that shape their expectations of what they should see which oftentimes conflicts with what they observe in their field placement causing what researchers coin as disequilibrium (Barry and Caravan, 2020). This disequilibrium, specifically with music PSTs, pushes them to focus on the interpersonal relationships between students and teachers rather than teaching as a profession involving a specific set of skills and knowledge. Disequilibrium has been seen in this study’s participant responses; however, their time away from the classroom may have given these PSTs more critical observation skills others may not have possessed before the Covid-19 shut down.

Researchers who study music education PSTs note how valuable the student teaching experience is to becoming a teacher (Abrahams, 2011; Kelly, 2015). For music PST retention, a positive experience during student teaching can help solidify a teacher’s commitment to the field of education. The student teaching field experience is an opportunity to learn and reflect on the experience of their CT, and the PST’s perspective of the CT’s classroom; it can also be valuable to the cooperating teacher to hear that perspective, even though the PST has limited knowledge and experience. Student teachers express that the most meaningful and beneficial reflective processes are those in which dialogue help advance the relationship between PST and CT to one of collaboration rather than master/apprentice (Stegman, 2007).

Despite these benefits, there are some significant issues with this model of preservice teacher field experience. The first limitation of co-teaching with PST is the lack of knowledge the PST brings into the experience; PSTs report a recognized lack of content knowledge that can create a barrier to a successful planning collaboration with their CT (Arndt and Liles, 2010). The lack of content knowledge is also a reason the co-teaching model can be so beneficial to the PST. Another limitation is the lack of planning time (Montgomery and Akerson, 2019). Given the appropriate atmosphere, most studies investigating the co-teaching field experience model have seen benefits for both the CT and the PST.

Teacher education institutions are beginning to focus on the student teaching field experience and using this time as a pivotal transitioning period for novice teachers. Institutions are using these cooperative and collaborative learning experiences to gradually transition PSTs into their first teaching jobs (Baeten et al., 2018). The co-teaching model can be utilized to achieve this goal. Co-teaching is a possible answer to unforeseen situations like the Covid-19 pandemic. For the PST in the field during the Covid-19 school closures were not in a co-teaching/team teaching circumstance, their experiences were either cut short or significantly altered for the worst (Daubney and Fautley, 2020). Another possible avenue for gradually transitioning PSTs is continued exposure to field-teaching experiences throughout the teacher preparation programs (Bartolome, 2017). Progressive field-experience starting early in a teacher’s preparation program could help prepare PSTs for any unforeseen situations during their full-time field-experience toward the end of their programs.

### Music Education Preservice Teachers’ Instruction and Learning Technological Activities

Before the Covid-19 school closures, music education had already begun to experiment with digital technology in conjunction with traditional music classroom activities (Crawford, 2017). In the context of synchronous and asynchronous music learning, music educators as early as the late 19th century began offering lessons and other musical opportunities *via* remote or distance learning platforms as basic as music lessons *via* radio transmission, TV broadcast, and now Zoom meetings (Hash, 2021). However, the pandemic forced many music educators who had not taken advantage of the growing music technology resources, to plunge deeper into the many techniques used to teach music remotely. Online technology can assist the music community through collaborative discussion opportunities for musicians and encourage a more in-depth focus on music learning and cognitive development (Johnson, 2017).

While the reasoning behind the implementation of music education digital learning varies by teacher, one obvious advantage is the ability to provide quality music educational experiences to those who may not be able to experience music opportunities (Cruywagen and Potgieter, 2005). Music students in remote locations can receive quality learning materials and resources through a distance learning paradigm using resources such as *The Sound Exchange* website, hosted by the Philharmonia Orchestra (Crawford, 2017), or the *Connect Resound* project in England (King et al., 2019).

The terminology synchronous and asynchronous were not been used so much before the pandemic and school closures, but the music education community was forced to address these various learning paradigm shifts. With these shifts, PSTs and CTs were required to adjust how they plan the day-to-day teaching activities, assessment strategies that integrate technology, and possible online distant performance opportunities (Mishra and Koehler, 2006). As seen through this study, PSTs were forced to deal with the various challenges that accompany technological music education integration, such as ensuring access to technology for all students, at home instrumentation, maintenance of privacy and security of online content, network connectivity, student internet activity monitoring, copyright laws, and student motivation and engagement *via* asynchronous and synchronous learning activities (Hash, 2021). These challenges may not have been as prevalent before the Covid-19 school closures, but they were present during the pandemic according to our participant responses.

Preservice teachers have been exposed to music technologies in the classroom whether in their own K-12 experiences or briefly through a music technology undergraduate course, but this exposure may not have prepared them for the implementation of musical technological pedagogical content knowledge (TPCK) needed (Mishra and Koehler, 2006). Remote learning stipulated during the Covid-19 school closures was necessary emergency teaching not a systematic implementation of a planned, organized, and well-designed distanced learning curriculum (Hash, 2021). Johnson (2017) suggested, to better prepare PSTs, in general education and music education, there needs to be a transference in pedagogical approach by teacher preparation faculty to better incorporate and support the application and maintenance of a technologically innovative pedagogical mindset. Through this pandemic music educators and PSTs have been forced to incorporate small group and the whole group videoconferencing, a focus on individual musicianship, lessons in music theory, history and culture, student creativity through composition and arranging with the assistance of music writing software, and other technological assessment strategies (Hash, 2021).

Technology may also allow new modes of instruction not previously possible including visualization of pitch content and musical notation. For example, classrooms often have large display monitors or smart boards to show digital tuning technology, chord diagrams or tablature, and play-along videos (Powell, 2019). Music-specific software is also on the rise and being further developed; in asking South African preservice music teachers about technology use, Gorgoretti (2019) specifically references composition software Finale, Sibelius, and Cubase; music creation, mixing, and editing software Garageband; music lesson software Music Ace; and digital music creation app Dance EJay. Further music-performance specific software, such as SmartMusic, has been studied as a means of performance assessment (Buck, 2008; Gurley, 2012; Perry, 2014). Distance learning technology may also facilitate connections between schools and professional organizations like The Sound Exchange, a website hosted for the music education activities of the Philharmonia Orchestra; *Berklee Shares*, a Music Improvisation interactive website; and online classroom software that unifies several messaging and social media platforms to help the online learning process (Crawford, 2017). Finally, some types of software may help students develop creative skills like audio manipulation and composition using notation. For example, Audacity is audio software allowing students to edit recorded audio, Noteflight is an online platform for the creation and sharing of music using traditional notation, SoundTrap is a browser-based audio editing and mixing software similar to GarageBand, and the Chrome Music Lab developed by Google allows students to create musical idioms using nonstandard notation (ideal for younger students).

Before the pandemic, many of these tools were used at the discretion of the teacher. But as schools were forced to move entirely online, these tools became central to all instruction. Many music educators struggled with this adjustment as music is typically communicated in live settings difficult to recreate online (Dye, 2016). Yet even before the pandemic, teaching and learning paradigms have changed as technology integration is part of ongoing curriculum reforms (Crawford, 2017). While the past impetus for such integration may have largely been due to “21st-century-skill” prioritization, the situation resulting from the Covid-19 pandemic has forced music educators to consider a tangible purpose behind efforts to use technology not just as a replacement tool for acoustic music-making, but as a means of revolutionizing ways of knowing and producing music (Mantie, 2020). Given this experience, preservice music teachers are poised to begin making new choices about what and how they teach music technology.

### Preservice Teachers and the University

Every teacher preparation program has its strengths and challenges that make them unique. Each state has different certification processes that make it difficult to standardize PSTs’ field experience requirements and procedures. However, research has shown that PSTs need continuous and early support in the organization and implementation of content-specific planning, teaching, and assessment (Baumgartner and Councill, 2019). For music education, pedagogy and methods courses are used to develop the necessary musical content skills needed in the various forms of music classrooms. Music education PSTs need a safe and cohesive environment where they can collaborate on lessons, observe advanced teachers, and participate in various forms of learning communities (Baumgartner and Councill, 2019). This environment must be achieved whether in the traditional teacher education programs or *via* online distanced learning programs.

Research comparing influential on-line teacher education with regular face-to-face classrooms allowed for an investigation into the on-line teaching and learning process, finding that in autonomous (or asynchronous) learning paradigms, group discussion among students was critical to student success and that adequate technology does not necessarily guarantee final learning product quality (Nishinosono, 2002). Regarding Music Teacher Education specifically, Montgomery et al. (2019) found that within a course using a Blended Learning Model (50% online and 50% face-to-face), the regularity of online access was significantly correlated with course achievement and that most (84.5%) student access instances occurred off-campus. The researchers hypothesized that some of this flexibility and regularity of access was in part due to the use of a flipped classroom design, in which the students led the instruction. Since the purpose of the face-to-face interactions was collaborative, whereas the asynchronous online content was more individually focused and involved learning of new material, the context was potentially a major impact on these findings compared to previous studies (Montgomery et al., 2019).

Video is a major component of asynchronous instruction which includes modeling appropriate teacher activity (Montgomery et al., 2019). In a review of literature on the use of classroom video in music education research, Bautista et al. (2019) found that classroom video had mainly been used to foster reflective practice of preservice and in-service teachers’ within the context of formal teaching programs. They suggest that, as in other education fields, music education might benefit from investigating the use of video as intervention practices or as a means of building content knowledge, classroom management skills, or fraternity among teaching colleagues (Bautista et al., 2019). Given that social distancing protocols in education often prohibit PSTs from visiting schools for field experiences during the pandemic, the use of pre-recorded or live video in distance teacher education is presumed to be a component worthy of study.

Preservice teachers needed more hands-on teaching time before their field experience and guided critical observation skills. PSTs schedules are filled with college courses as well as responsibilities to their CTs and their K-12 students. Many teacher education programs require PSTs to observe practicing teachers, but often these observations do not ensure reflection upon the music teaching and learning (Barry and Caravan, 2020). The thoughtful observation skills needed to learn from observation and transfer the pedagogical techniques seen are not always discussed in methods courses for undergraduate teachers (Barry and Caravan, 2020). Courses, such as music teaching seminars, have the possibility of being the environment that will bridge the gap between the idealistic university setting and the realistic K-12 school music classrooms PSTs experience (Baumgartner and Councill, 2019). Through this pandemic, music teacher preparation programs can see where the gaps fall when transitioning undergraduate students to student teaching to eventual independent teaching.

### Preservice Teachers’ Challenges and Covid-19 Recent Research

At the time of this writing, little had been published about the experiences of PST during the pandemic. In response to initial quarantine guidelines, both college courses and K-12 education moved to online platforms. PST had to exit the classroom and it was often suggested they observe videos of classrooms and present lessons to their peers using online videoconferencing software. While “peer teaching” is a staple of earlier PST practice teaching experiences, it is not a substitute for field experience with K-12 children. Therefore, it is incumbent on teacher preparation programs to examine the various modalities of online distance learning and model the best alternatives to in-person learning (Kim, 2020). Among those options that Kim identified were peer-teaching among PSTs in an asynchronous online setting, where PSTs can receive feedback not just from the faculty observer or CT, but also PST peers. While limitations of synchronous online learning include a potential lack of direct feedback to the children and an inability to utilize collaborative learning strategies or hands-on activities, the most advantageous scenarios for young children are still synchronous and in small groups (Kim, 2020).

Meanwhile, Daubney and Fautley (2020) describe the challenges and opportunities for specialist music educators to reach a generalist audience through online, individualized learning. Their main concerns focused on the technical disparities among different economic classes of students, which may be even more apparent when we return to in-person schooling. As this is also true of education in Georgia (Shearer, 2020), the location of the placement school may hinder fully intensive, extensive, and immersive culminating field experience. In the case of the Spring 2020 semester, placements were already set and students were in mid-experience when they were abruptly shifted online; there was not enough time to reflect on whether an online experience was even feasible in their school’s location or their own home, and what technical or structural limitations would occur. Hence, in this study, we were very interested in how PSTs and CTs adapted in a variety of contexts across Georgia.

Teacher preparation program professors must move toward a comprehensive and grounded view of the necessary pedagogy of online education (Carrillo and Flores, 2020). The pandemic has forced teacher preparation faculty to go beyond emergency online practices and incorporate evidence-based approaches to online music teaching and learning (Carrillo and Flores, 2020; Hodges et al., 2020). Through this study and others, the PSTs aware of the various pre-existing music applications for music notation, music performance, listening and watching music, recording and mixing music, and musical assessment has increased (Lehimler, 2019). However, despite this awareness, the ability of PSTs to integrate these applications into a real-life day-to-day classroom lesson is lacking. Lehimler (2019) shows that the application of instrumental education applications for musical performance and assessment has been mostly used for purposes of professional development and not pedagogical technique.

The purpose of this study was to understand changes to music education PSTs experiences during the initial months of the pandemic. In early April 2020, we quickly created a survey for music education PSTs in the state of Georgia to collect data on their level of involvement with planning, instruction, and assessment at their student teaching K-12 placement. Additionally, we were interested in what types of musical activities music teachers chose to implement in the virtual setting, technology used, and PST perceptions of student learning and their learning in this virtual environment. Specifically, the research questions were: (1) How and in what ways were PSTs involved in planning, instruction, and/or assessment synchronously and asynchronously after school closures? (2) In what subdomains (performance, music theory/ear-training, etc.) were instruction and learning activities? (3) What challenges and solutions did PSTs report related to Covid-19 closures?

## Materials and Methods

Within one month of closures, we developed a survey to find out what the PSTs were encountering in the remaining months of their internship, the survey can be found in Appendix A at the end of the manuscript. Questions included PST demographics, school placement demographics, offered music programming, PST involvement in planning, instruction, assessment for music performance, theory/ear-training, music history, and music composition. PSTs were also asked open-ended questions about challenges they overcame and any surprises they encountered in their students’ learning and their learning. After obtaining Institutional Review Board (IRB) approval, the survey was distributed to music education professors involved infield experience at all 23 Georgia universities with music education degree programs. In total, 29 professors were contacted and asked to forward the survey invitation by email to their music education PSTs. Though the exact number of PSTs completing the final semester of internship during spring of 2020 is unknown, 176 students took the GACE music education content exam (required state licensure exam) in the Fall 2019 and Spring 2020 school year. We approximate that about two-thirds of those (116) were Spring 2020 student teachers.

Thirty-seven responses were received which is approximately a 32% response rate. The survey responses were compiled into a Microsoft Excel file and then imported into MAXQDA (VERBI Software, 2019) for coding. Participants’ ages ranged from 21 to 25 with the majority (22) of the participants being 21 or 22. There were 21 female participants and 16 male participants. The school settings of the participants were categorized into the setting (10 rural, 21 suburban, and size urban) as well as grade levels (five Elementary, 18 Middle, 12 High, one Middle/High combination, and one K-12 school).

The participants provided the approximate school population (*M* = 1,505, ranging from 113 to 3,800) and the total number of students enrolled in the music courses the CT was teaching (*m* = 287, ranging from 60 to 1,500). After categorizing the participants’ placement schools, the remaining information was further categorized. The information from the surveys was uploaded into the software and the questions were identified as variables and codable data. If the survey question was a drop-down choice, the answers were labeled as nominal variables (e.g., school setting rural/suburban/urban), and all short answer questions were separated to be coded. Of the 38 questions, 15 questions were dedicated to the participants’ placement school demographics and what specific music classes were offered, and which the participants were responsible for teaching. The full survey instrument is available in the supplementary material (see Appendix A).

Saldaña (2021) thematic coding was used to create the initial codes from the short answer survey questions. Each short-answer question was read and coded. The coding system was created using *in vivo* coding incorporating the participants’ words. Once the codes were generated, through the use of consolidated meaning research the principal researcher (MT) used the codes created with the assistance of the second author (MN) to create the categories (Saldaña, 2021). The initial large categories consisted of: challenges, student assignments, and activities, positives, negatives, parents/family, university work/responsibilities, support, student engagement, videos/recordings, cooperating teachers, technology, and lesson plans. The most frequent categories were negatives, positives, university work/responsibilities, and technology. The categories were recategorized later in an attempt to eliminate any unintended bias. These categories are used to generalize the implications for future music teacher education and music technology pedagogy explored in the discussion portion.

The categories led to the themes used to address each research question (Saldaña, 2021). The common themes highlighted through the coding and later categorization process helped form the content of the discussion section. Focus coding was then used with cross-referencing to put the data through a second cycle analytic process (Saldaña, 2021). Within the MAXQDA software, the codes were categorized and clustered again, and then cross-referenced with the numerical variables. This second analytical cycle was employed to assist in the discussion of the research questions.

## Results

The responses were received between April 14th and May 12th, 2020. During these dates, the state of Georgia was still under the government-imposed shelter-in-place. Twenty-one of the participants noted that they were not given access to their placement schools’ online teaching platform. Despite these PSTs not being allowed access, 17 of those 21 were still involved in both asynchronous and synchronous music performance learning. When we received the first response, there were 12,193 confirmed Covid-19 cases and an additional 20 people died on this day due to the virus. On May 12th when we received the final response, Georgia had accumulated 32,750 confirmed Covid-19 cases and an additional 24 people died on this day (United States Census Bureau, 2020).

Georgia residents had first been banned from large group gatherings. The school systems were ordered to stop all in-person learning and switch to distant online learning completely. Childcare facilities were shut down to accommodate the growing health concerns. School boards were meeting virtually to decide on testing. Public schools were supplying students with meal relief services from meal deliveries and stations. Universities were forced to shut down face-to-face classes and also move all learning to digital platforms.

The participants were asked how long had passed between the school closures and when they first had contact with their CT; 10 participants chose the answer “contacted immediately,” eight chose “contacted within a few days,” four chose “contacted after a week,” three chose “contacted after two weeks,” nine chose “never allowed to participate after closures,” and three chose “still waiting to be contacted.” Of those 10 who said they were contacted immediately post school closures, only three were allowed access to their placement school’s online learning platform.

After the closure participants were engaged with several music activities; creation of additional instruction and help videos for students, virtual materials and assignments for students, music theory worksheets, assessed students “passing off” selected repertoire, participated in music learning games with students, assigned and assessed recorded videos of student work, and attended Zoom class sessions.

The participants were asked what challenges they faced after the school closure, personally and in their experience with virtual student teaching. See Table 1 for quoted examples related to engagement by the K-12 students taught by the PST and how that affected the PST and CT. PSTs reported having difficulty with different types of learners in the classroom, issues with self-guided learning for students and PSTs With limited classroom time they noted the need for lesson plans that are easily transferred from face-to-face to virtual, the need for more planning time. Taking the pandemic circumstances PSTs mentioned issues with personal coping skills, students without instruments at home, lack of student engagement, and a lack of motivation from PSTs and students. Time effectiveness in lessons and self-learning posed more issues for the PSTs along with a lack of support from CT and university faculty, and internet instability. The K-12 students’ lack of achievement on assignments, other classroom teachers giving students too much work, not doing anything after the school closure, and missing face-to-face interactions were some of the other challenges the PSTs faced. These answers were given by both PSTs with access to their placement school’s virtual teaching platform and those not given access.

**Table 1:** Participant comments regarding student engagement.

*“Many of our students are not doing well in their theory assignments. I am surprised that more of them are not joining the online help sessions”*
*“They can actually learn. It's not impossible like I originally thought. Now it is ineffective because a lot of students do not participate”*
*“Many students did not complete any of the online assignments, even the normally attentive and advanced students”*
*“Some of them have not completed the assignments to the best of their abilities that I’ve seen them do prior to going to online learning”*
*“Students (and the teachers, myself included) have lost the drive to continue. It is now May while doing this survey, and it has been roughly a week without any significant work done in the classroom to my knowledge. It has mostly been worksheets. Most of the higher-level classes are also senior and juniors. Senioritis has been rough when you do not get a prom, graduation, final concert, final musical theater show…”*

The open-ended responses were also categorized as either positive or negative. It is interesting to note that there were 45 codes categorized as negative and 35 codes categorized as positive. Negatives included a lack of motivation, students not doing well on the assignments, students being overwhelmed with other work, lack of engagement, missing face-to-face interactions, and a feeling of “senioritis.” Despite the outwardly negativity of the pandemic, it is fascinating that there were almost as many positive codes as there were negative codes, insinuating a neither overtly positive or negative field experience for the PSTs during the school closures.

Positives were that the students felt comfortable enough to reach out to PSTs for help, students remembered things from their face-to-face lessons, students were technologically equipped, students showed each other respect in virtual lessons, some students were more excited about learning, and students were doing well on assessments.

The PSTs used a variety of technology resources to help in student engagement of musical activities, including BandLab, FlipGrid, Foliotek, Google Classroom, Incredibox.com, Musictheory.net, Tone Savvy, Shedthemusic.com, Quaver, and various video conferencing software. The participants were asked to give a list of technologies used in planning, instruction, and assessment.

Some participants mentioned their relationship or interactions with their CT, some positive, and some negative. PSTs mentioned co-teaching successfully virtually with their CT, having collaborative planning meetings with their CT, continued a great relationship and communication with CT after the closure, CT continuing to offer advice and mentor the PST through the closure and virtual experience, and the PST being invited to join and participate in virtual faculty meetings alongside CT. PSTs also reported CTs not asking for help after the school closures and the need for more collaboration with their CT to make teaching easier. These responses were given by participants despite not having a survey question asking directly about the PST and CT relationship change. Some of the responses involving CTs were from the question that asked the PST about their challenges using technology, positive and negative surprises to the online student learning format, PST descriptions of their synchronous and asynchronous teaching involvement, and PST descriptions of their involvement in teaching, learning, and assessment of the K-12 students. PSTs reported both negative and positive changes in the relationship between PST, CT, and students. Negative changes included lack of support, non-engagement, challenges progressing, and lost teaching time. Positive changes included increased support given by the CT and more time to plan with their CT.

Regarding the participant’s engagement in their teacher education programs post-school closure, they noted assignment changes including watching videos of other teachers being helpful, spending time filling out job applications, and discussion boards and Zoom calls with other PST being engaging and helpful. Participants mentioned online work assignments and portfolio assignments but did not make any comments about their usefulness or helpfulness. PSTs noted that they feel teacher education programs should have a stronger focus on virtual lesson plans specific to the field of music education and address communication breakdowns between PST and CT to plan for the unforeseen.

The final questions of the survey asked the PSTs about their journey during the Covid-19 school closures and for any additional comments. PSTs mentioned feeling their teaching did not change since the closure, they missed their students, they felt their progression was stifled by virtual learning, and they missed teaching time in the classroom. “It made me stop teaching, the platform for student’s assignments was already made, so my job became teaching the students on my own while my mentor uploaded assignments to the online course. Afterward, I’ve had no involvement in the classroom” (Participant 35), “I have struggled with having to give up my final performances and not getting closure with my kids. I also lost my great-grandmother to Covid-19, which has been very hard” (Participant 24). PSTs also mentioned having time to practice secondary instruments, volunteering their time elsewhere, the allowance to see the grading system in action, more focus put on their creative teaching techniques, the positive effects of their face-to-face interactions before the closure, building trust in themselves and their teaching abilities, the importance of built relationships with CT and students, and heightened awareness to their self-development as a teacher. “Developing my skills has been limited ‐ I have found more time to practice my instrument” (Participant 7), “I have volunteered to help deliver meals with the school teachers in my home community, but I was not invited to help with online learning in the community in which I was student teaching. My learning has shifted to online, though I have mostly only had to work on finishing portfolios and other paperwork for my certification” (Participant 24), “I’m learning a lot by practicing secondary instruments and just trying to use my time as effectively as possible to learn as much as I can, but I’m not teaching at all, and I miss it” (Participant 8).

We did explore whether responses differed systematically based on participant demographic information and school placement setting. This analysis did not find any meaningful differences between sample subgroups likely due to the small sample size. However, again important to note the similar number of negative (45 codes) and positive codes (35 codes).

## Discussion

The beginning of the Covid stay-at-home orders challenged music teachers, preservice music teachers, and students to figure out how to make music without the joy and convenience of face-to-face interactions. As faculty guiding PSTs, we sought to understand how this monumental change in teaching impacted the use of technology, relationships between mentor teachers and PSTs, university support for PSTs, and the kinds of activities PSTs were able to engage in. A unique aspect of this study is the timeframe during which we collected data. In the United States, schools shut down in mid-March. Our data collection was completed within the first 2 months of the pandemic, showing a wide variety of levels of engagement among PSTs in their student teaching placements.

### PSTs’ Engagement in Teaching

Almost 60% of PSTs were contacted by their CT within 1 week of school closures. Likely, most universities and schools did not have plans in place that provided policies or guidelines for what to do in the event of extended school closures. This may have caused some CTs to believe that the universities would be entirely responsible for the PSTs, accounting for the slow response rate of the remaining 40% of CTs.

The PSTs were placed in rural, suburban, and urban settings. Internet access and speed were not equal in all parts of the state, with schools in rural areas particularly lacking access for students (Shearer, 2020). Therefore, the teaching activities the PSTs engaged in largely depended on the kind of instruction their host school was providing. For online asynchronous settings, they created introduction videos of new concepts or songs, created remedial instructional videos, and assessed individual band students’ performances. In online synchronous classes, they were generally observed because the PSTs did not have access to teach in the district learning management system, or because the mentor teacher was still developing their approach to teaching online. Our data did not allow us to determine if the reduction in face-to-face or even online teaching negatively impacted PSTs’ commitment to teaching music long-term (Kelly, 2015). Future research should follow up with PSTs impacted during the Covid-19 pandemic to investigate potential bearings on in-service teacher retention.

### Relationships Between CTs and PSTs

Though we asked no questions about the relationships between CTs and PSTs, the respondents made comments that support the research literature. The relationship between a student-teacher and CT is a key component of the student teaching experience (Bell and Robinson, 2004; Denis, 2017; Lejonberg et al., 2018). Student teaching experiences often include variations of co-teaching, with the PST and CT exchanging roles as primary and supportive instructors or splitting students into work groups (Kim, 2019; Montgomery and Akerson, 2019; Zach, 2020). In this early stage of school shut-downs, PSTs were rarely able to participate in this important developmental skill. Instead, they were generally limited to “off-line” tasks, such as creating materials for the CT to distribute to students. However, several PSTs stated that many aspects of mentoring were able to continue. “During our Zoom meetings, my co-op [CT] and I would alternate leading the review of the music theory worksheets.” (Participant 37); “My cooperating teachers have been incredibly supportive, and I am very appreciative of that” (Participant 19); “I plan with my cooperating teachers on Zoom and help create materials on my own as well… I have worked on assessment with my cooperating teachers on Zoom as well as providing feedback on my own” (Participant 19); “Though my cooperating teacher and I would sit down and plan the structure and lessons for the class, I was entirely in charge of instructing and assessing the class, he would assess me after the class ended” (Participant 32); and “I had a really great overall experience. After the Covid-19 closure, I maintained a close proximity to the collaborating teacher and felt like I was pretty involved with online learning as much as I could given the online curriculum” (Participant 31).

### Recommendations for Teaching and Learning

Pre-Covid articles about the role of technology in music education have often cited increased accessibility as an important reason to expand technology in music education (e.g., Cruywagen and Potgieter, 2005). Therefore, it was interesting to observe that when the majority of music education was thrust into a technological framework, many unanticipated obstacles arose. Even more than 6 months after data collection and access to internet, local infrastructures in Georgia (population 10.6 million) still struggle to provide equitable access for students (Shearer, 2020). Teacher preparation programs are working to meet PSTs’ recommendation to include “how to teach in a virtual environment” (also, Kelly, 2015; Dye, 2016; Daubney and Fautley, 2020; Kim, 2020).

Early in the pandemic, teachers and PSTs did not have time to consider the move to virtual instruction as an opportunity for change. They had as little as a few days to consider their technology skills and equipment, the learning priorities for their students, and the equipment in their students’ homes. Also, many schools were on constantly evolving stay-at-home plans, which made long-term planning nearly impossible. Alternative approaches, trying to recreate traditional ensembles and classes would have been able to explore the technology used in electronic ensembles such as iPad ensembles (e.g., Williams, 2014) or creating composition projects (Joseph, 2020). Teacher preparation programs should incorporate more distance learning in all aspects of pedagogy from available technologies, lesson plan development, and execution, to impact on learning outcomes and issues of equity among different student populations and school settings.

### Challenges Typical to ST Exaggerated by Covid, Unique to Covid

The PSTs identified challenges that were consistent with traditional student teaching but likely exacerbated by the shutdowns and other challenges unique to this situation. The PSTs commented that they wish they had more time for planning and their professional growth, which is consistent with concerns about time in traditional student teaching (Conway et al., 2005). Main concerns included instruction delivery, engaging learners, and the lack of time to prepare. Teaching different types of learners is challenging even in the most ideal teaching contexts. Here, some PSTs specifically identified this concern with regards to technology use during a synchronous lesson *via* Zoom:

“The [online] classrooms are very overcrowded and that makes it difficult for classroom management but also to accommodate the different types of learners in the room. The biggest way that I feel helped me was to just be honest with the students so they would be honest with me. I made it clear that if they were not understanding something all they had to do was raise a hand either in the air or in their lap if they didn’t want to make it known to the whole room. When I saw hands I repeated what I was explaining using a different way. So drawing instead of speaking. Or moving instead of singing” (Participant 17).

Likewise, the PSTs had more difficulties than usual in engaging students in learning, as reflected in the comments in Table 1, located at the end after the reference list.

It quickly became apparent to PSTs that the move to online teaching was not as simple as just re-writing lesson plans for recorded video or synchronous video (Kim, 2020). Young children did not have their own instruments at home, and many of the band and orchestra school and personal instruments were locked up in the closed schools. Student engagement online was even more challenging than in classes: “Not every student has an instrument, and therefore we cannot give many performance-based assignments or instruction. My teachers are largely doing this through video tutorials for students who do have instruments at home” (Participant 3); and “I can’t see students’ faces and I miss them so much! I’m not able to teach the content I want, especially since not every student has an instrument…” (Participant 3).

### Impact on University Support

In addition to CTs supporting PSTs, the university supervisor is the other primary mentor during student teaching (Denis, 2017). The sudden shutdown of schools meant university supervisors needed to provide supplemental experiences for the PSTs, for them to continue their professional growth. These included creating Professional Learning Communities (Battersby, 2019), compiling videos for PSTs to watch, providing support for job applications, and guidance on developing portfolios. One participant elaborated:

I wish I felt a little more supported by my university faculty, and like they cared about me as a person rather than just as a student; I do better when I have specific assignments, due dates, and expectations, but my professors have left it completely up to us how to use this time. I don’t know if I’m meeting their expectations or not, which makes it a little stressful. I wish we had more guidance from our university faculty. I’ve been seeking out opportunities for professional development wherever I can find it since it isn’t coming from my professors.

In order to support PSTs during distance teaching, specifically in this sense during the online emergency instructional-setting, both the CTs and university supervisors and faculty members should innovate their teaching (including PST supervision) with Information and Communication technologies (ICT; Schildkamp et al., 2020). Despite the shut down due to the pandemic, many innovative technological techniques have been utilized both in higher education and K-12 schools; however, what will happen when schools go back to “normal”? It is the hope of all the education world that the technological advances used during the pandemic will remain.

## Limitations

This study was conceived, and the survey was dispersed at the beginning of the pandemic and immediately after the school closures. With this consideration, the small sample size of responses is understandable. However, this small sample size does not allow for generalizability. The small sample size did not allow for analysis and conclusions related to student demographics and school settings. Future research should investigate these issues specifically as related to technology use. The research team chose to only accept responses for 2 months, which may have limited response rate. It is also possible that at the time of the responses, some PSTs had not yet been contacted by their CT but may have received communication later. A final limitation of the study is the population from only one state. It would prove advantageous if this study is performed again and includes all states music education teacher programs.

## Recommendations For Further Research

Through the themes discovered in this research PSTs, specifically in the music education field, need more time in their university methods and pedagogy classes focused on field-specific learning activities, lesson planning and implementation, assessment strategies, and music-specific technological application. University professors must take more time to discuss, observe, and collaborate with PSTs on how to collaborate with their CTs on lessons, learning activities, and integration of technological resources. Based on previous literature and this study’s participation responses, PSTs are aware of the music technology available to them but are oftentimes unsure of how to apply them in a real-life classroom setting or *via* distance learning platforms. Early application of these skills for PSTs must take place before their student teaching field experience if they are to be prepared for their successful integration.

The pandemic and eventual school closures forced music educators to invent and implement emergency distance learning lessons and performance opportunities. However, with the eventual integration of K-12 asynchronous and synchronous learning, for music educators to remain relevant and part of the daily curricular make-up of their schools, they must adapt. The traditional music classroom paradigm is shifting to make room for technological advancement and music educators on all levels, university, and K-12, have to shift or be left behind. Further research into music education synchronous and asynchronous content and application is needed to fully help prepare new teachers for their first jobs. Research into the collaboration between music education PSTs and CTs will help university faculty members make the best-informed decision on PST field experience models and the curriculum for their methods and pedagogy courses.

## Data Availability Statement

The raw data supporting the conclusions of this article will be made available by the authors, without undue reservation.

## Ethics Statement

The studies involving human participants were reviewed and approved by Human Resource Protection Program University of Georgia. The patients/participants provided their written informed consent to participate in this study.

## Author Contributions

MN, LS, RA, AK, and AF conceived the study and designed the survey. RA formatted and administered the survey and handled related permissions. MT analyzed the data and wrote the initial draft under the supervision of MN. All authors contributed to the article and approved the submitted version.

### Conflict of Interest

The authors declare that the research was conducted in the absence of any commercial or financial relationships that could be construed as a potential conflict of interest.
