# Supplementary material for: Online Involvement for Georgia Student Teachers During Covid-19
Source: Front Psychol. 2021 Jun 3;12:648028. doi: 10.3389/fpsyg.2021.648028 (PMC8209252; doi:10.3389/fpsyg.2021.648028)
Supplement: Supplementary file 1 [file Data_Sheet_1.pdf]

Block 3

UNIVERSITY OF GEORGIA

CONSENT LETTER

Online Involvement of Georgia music student teachers during Covid-19 closures

Dear Participant,

We are music education professors from the University of Georgia and would like to invite you to participate in a research study entitled "Online Involvement of Georgia music student teachers during Covid-19 closures." The purpose of this study is to collect information about the experiences of student teachers completing the remainder of their student teaching or practicum online due to school closures. We have asked your music education faculty to forward this survey to you.

You're eligible to be in this study because you are completing your student teaching or practicum during the spring of 2020.

Your participation will involve completing an online survey answering questions about your access to online platforms, internet access, and your engagement in planning, teaching, and assessment. The survey should take between 20-30 minutes. No identifiable information will be collected outside of the IP address if you are using a personal computer, tablet, or home internet. The identifiers will be deleted as the first step in analyzing the data.

Participation is voluntary. You can refuse to take part or stop at any time without penalty. Your decision to participate in this study will have no impact on your participation in or completion of

your university program. Your decision to take part or not to take part in the research will not affect your grades or class standing.

We do not anticipate any questions making you uncomfortable, but if there are, you can skip these questions if you do not wish to answer them. Furthermore, you may choose to stop at any time or choose not to submit the data at the end of the survey.

This research involves the transmission of data over the Internet. Every reasonable effort has been taken to ensure the effective use of available technology; however, confidentiality during online communication cannot be guaranteed

Anticipated benefits include you having an opportunity to reflect, analyze, and synthesize learning as well as think about others in different but similar situations. Your responses may help teacher educators and music educators gain more insight to the many different situations and opportunities available for music teaching during the COVID-19 closures.

The information may be shared after the indirect identifiers have been removed with other researchers for data analysis to improve practicum and student teaching experiences within higher education without additional consent.

Furthermore, your collective ideas for lesson planning, online instruction, technology use, and assessments will be shared through presentations and publications.

If you are interested in participating or have questions about this research, please feel free to contact **Rebecca Atkins** at *499-470-0328, rlatkins@uga.edu or* **Alison Farley** at *alpfarley@uga.edu*. If you have any complaints or questions about your rights as a research volunteer, contact the IRB at 706-542-3199 or by email at IRB@uga.edu.

By answering Yes and clicking next, you are giving your consent to participate in the study.

I am currently student teaching

Yes

No

Block 2

Age

[ ]

Gender

Male

Female

[ ] other

prefer not to say

What type of setting does your placement school represent?

urban

rural

suburban

What is the setting of your placement? (check all that apply)

elementary

middle school

high school

Pk-12 (or K-12)

[ ] other

Check all grade levels at your school

PK

K

1

2

3

4

5

6

7

8

9

10

11

12

Approximate number of students in this school

Approximate number of students for whom your cooperating teacher(s) are responsible for music instruction

How many hours per week were you assigned to your student teaching placement prior to the school closings? (In class and after school rehearsals)

What music courses are available at your school during the school day?

general music
music appreciation (if different from general music)
choir
band
orchestra
guitar
theory
technology
keyboard
applied lessons
musical theater
show choir
jazz band
other(s)

What music courses are available at your school outside the school day?

general music
music appreciation (if different from general music)
choir

band

orchestra

guitar

theory

technology

keyboard

applied lessons

musical theater

show choir

jazz band

[ ] other

Which courses were a part of your responsibility in your teaching assignment?

general music

music appreciation (if different than general music)

choir

band

orchestra

guitar

theory

technology

keyboard

applied lessons

musical theater

show choir

jazz band

[ ] other

Default Question Block

Do you have the internet capabilities from home to continue in online teaching?

Yes

No

Yes, but internet service is not always reliable

Were you given access to your school's online teaching platform?

Yes

No

Once school closure was announced, how much time passed before you worked with your cooperating teacher again at your placement? (don't include spring break week if applicable)

I was requested to participate:

Immediately

within a few days

after 1 week

after 2 weeks

after 3 weeks

still waiting

never invited or allowed to participate.

Are you engaged in Music Performance teaching with students' main instrument? (check all that apply for both synchronous and asynchronous)

	Planning (with teacher)	Instruction (to students)	Assessment (of students)
Synchronously	☐	☐	☐
Asynchronously	☐	☐	☐

Please provide a description of an activity you are engaged in for each box checked for planning, instruction, and assessment.

Are you engaged in Music Performance teaching with students that involve an instrument that is NOT the student's main instrument? (check all that apply for both synchronous and asynchronous)

	Planning (with teacher)	Instruction (to students)	Assessment (of students)
Synchronously	☐	☐	☐
Asynchronously	☐	☐	☐

Please provide a description of an activity you are engaged in for each box checked for planning, instruction, and assessment.

Are you engaged with music history teaching with students? (check all that apply for both synchronous and asynchronous)

	Planning (with teacher)	Instruction (to students)	Assessment (of students)
Synchronously	☐	☐	☐
Asynchronously	☐	☐	☐

Please provide a description of an activity you are engaged in for each box checked for planning, instruction, and assessment.

Are you engaged with music theory teaching with students? (check all that apply for both synchronous and asynchronous)

	Planning (with teacher)	Instruction (to students)	Assessment (of students)
Synchronously	☐	☐	☐

	Planning (with teacher)	Instruction (to students)	Assessment (of students)
Asynchronously	☐	☐	☐

Please provide a description of an activity you are engaged in for each box checked for planning, instruction, and assessment.

Are you engaged with music ear-training with students? (check all that apply for both synchronous and asynchronous)

	Planning (with teacher)	Instruction (to students)	Assessment (of students)
synchronously	☐	☐	☐
asynchronously	☐	☐	☐

Please provide a description of an activity you are engaged in for each box checked for planning, instruction, and assessment.

Are you engaged with an activity with students that does not fit the performance/history/theory/ear-training categories? (check all that apply for both synchronous and asynchronous)

	Planning (with teacher)	Instruction (to students)	Assessment (of students)
synchronously	☐	☐	☐
asynchronously	☐	☐	☐

How would you label/categorize this activity outside of performance, theory, history

Please provide a description of an activity you are engaged in for each box checked for planning, instruction, and assessment.

Give example(s) of something that has surprised you about your students learning (in a good way) in the online format.

Give example(s) of something that has surprised you about your students learning (in a not so good way) through online format.

Block 1

What challenges have you found using technology and in what ways have you worked through those challenges?

What challenges have you had in general and in what ways have you worked through those challenges?

What has changed about your own teaching and learning through the closure of schools and online platform teaching and/or learning.

Please list any new technology tools you have learned that you want shared with others. Briefly describe the application and how you use it. Also explain if this technology was used for teacher organization or student learning.

What activities have you engaged in to complete the requirements for your student teaching course for your university to replace the instruction time lost due to Covid-19 school closures? (Please list as many as you can).

Please write any other comments you wish to share about your student teaching situation completion.

Powered by Qualtrics
